# Supplementary material for: Empirical methods for controlling false positives and estimating confidence in ChIP-Seq peaks
Source: BMC Bioinformatics. 2008 Dec 5;9:523. doi: 10.1186/1471-2105-9-523 (PMC2628906; doi:10.1186/1471-2105-9-523)
Supplement: Additional File 1 — A variety of html documents from the USeq web site detailing the available applications, their best usage, output file type descriptions, command line menus, etc. [file 1471-2105-9-523-S1.zip › USeqUserGuides/JavaDoc.zip!/JavaDoc/util/bio/parsers/package-summary.html]

util.bio.parsers


|  |  |  |
| --- | --- | --- |
| **Overview** \| **Package** \| Class \| **Use** \| **Tree** \| **Deprecated** \| **Index** \| **Help** | |  |
| **PREV PACKAGE**   **NEXT PACKAGE** | **FRAMES**    **NO FRAMES**     **All Classes** |  |


---

## Package util.bio.parsers

Parsers and extractors, fasta, quality, text, gff....

**See:**
  
          **Description**

| **Class Summary** | |
| --- | --- |
| **ADFDataBaseSplitter** |  |
| **BaseCount** |  |
| **BlastCompompositeGenerator** |  |
| **BlastParser** | Copied from the BioJava Cookbook - Keith James. |
| **Cap3Parser** |  |
| **DrosGeneCollFileParser** | Parses and extracts a unique set of gene names from a drosophila gene collection file. |
| **Fasta** |  |
| **FetchGenomicSequences** | Fetches the genomic sequences for a list of regions. |
| **FetchSeqsQualsByName** |  |
| **MultiFastaParser** | Extracts sequences and headers out of multi fasta files. |
| **ParseDecodeGenotypeData** | Parses a directory of joined decode data files, ie 'chr10 358624 3 0 0.992874 cnv1071p9' chr,pos,#A,#B,1-pval,SNPName. |
| **QualityFileParser** | This class will read in combine quality files associated with raw sequencing results, skips blank lines, parses off the > character. |
| **RefSeqAnnotationParser** | Parser for pulling gene annotations into a UCSC table format for Sodalis. |
| **RipInparanoidResults** |  |
| **RipMGIOrthologyResults** |  |
| **ShiftPositionsInUCSCGeneLines** | Shifts the bp position of UCSCGeneLines matching particular chromosomes.Good for shifting coordinates after making a composite mock chromosome from unassembled sequences/ scaffolds. |
| **SynonymMatching** |  |
| **UCSCGeneLine** |  |
| **UCSCGeneLineComparator** |  |
| **UCSCGeneLineComparatorScore** | Sorts by score |
| **UCSCGeneModelTableReader** | Parses a UCSC gene model table file (either refSeq or refFlat), use table options for #hg17.refGene.name hg17.refLink.product hg17.geneName.name hg17.refSeqSummary.summary... |

## Package util.bio.parsers Description

Parsers and extractors, fasta, quality, text, gff....

---


|  |  |  |
| --- | --- | --- |
| **Overview** \| **Package** \| Class \| **Use** \| **Tree** \| **Deprecated** \| **Index** \| **Help** | |  |
| **PREV PACKAGE**   **NEXT PACKAGE** | **FRAMES**    **NO FRAMES**     **All Classes** |  |


---
